# Can enlightenment be traced to specific neural correlates, cognition, or behavior? No, and (a qualified) Yes

**DOI:** 10.3389/fpsyg.2013.00870

**Published:** 2013-11-22

**Authors:** Jake H. Davis, David R. Vago

**Affiliations:** ^1^Department of Philosophy, City University of New York Graduate CenterNew York, NY, USA; ^2^Functional Neuroimaging Laboratory, Department of Psychiatry, Brigham and Women's Hospital, Harvard Medical SchoolBoston, MA, USA

**Keywords:** mindfulness, meditation, awareness, enlightenment, consciousness, neurophenomenology

## A neuroscience of enlightenment?

The field of contemplative science is rapidly growing and integrating into the basic neurosciences, psychology, clinical sciences, and society-at-large. Yet the majority of current research in the contemplative sciences has been divorced from the soteriological context from which these meditative practices originate and has focused instead on clinical applications with goals of stress reduction and psychotherapeutic health. In the existing research on health outcomes of mindfulness-based clinical interventions, for example, there have been almost no attempts to scientifically investigate the goal of enlightenment. This is a serious oversight, given that such profound transformation across ethical, perceptual, emotional, and cognitive domains are taken to be the natural outcome and principle aim of mindfulness practice in the traditional Buddhist contexts from which these practices are derived. If short-term interventions as short as a few sessions are now beginning to produce neuroplastic changes (Tang et al., 2010; Zeidan et al., 2010; Xue et al., 2011), it may be that even in secular contexts, practitioners are already developing states and traits that are associated with progress toward enlightenment. In order to carefully assess the potential effects of meditative interventions it is of singular importance to ask whether enlightenment can be traced to specific neural correlates, cognition, or behavior.

## No: enlightenment needs specification

Unfortunately, the first answer to this question is “No.” The term ’’enlightenment” is an extraordinarily imprecise construct. Using the term enlightenment or even the term more native to Buddhist traditions, “awakening” (*bodhi*), as if it referred to a single outcome either privileges one conception over others or else assumes that there is some commonality among the traditional goals of diverse contemplative traditions. There are deep disagreements over the nature of the goal between and even within various Buddhist schools. Scientific investigations cannot assume that there is any commonality among the transformative changes referred to as “kensho,” “stream entry,” “realizing the nature of mind,” and so on, that various Buddhist traditions take as various stages of awakening. Empirical investigations of these constructs can only proceed with reference to the specific psychological and behavioral outcomes described in the native discourse of a specific tradition (see Lutz et al., 2007). Because we do not have space here to offer detailed examinations of the theoretical context and neurobiological characterization of enlightenment as it is specified in multiple Buddhist traditions, we focus here on one specific example, that of the Theravāda Buddhist tradition of the Burmese meditation master Mahasi Sayadaw. Nonetheless, by applying more generally the approach we adopt here for this tradition, claims for commonality or differences between psychological states and traits associated with various traditional goals can be operationalized as testable hypotheses.

While some Buddhist traditions understand the goal of *nibbāna* just as a cessation of unwholesome mental states, Mahasi Sayadaw maintains that *nibbāna* “can be seen inwardly as the cessation of all phenomena” (Sayadaw, 1992). This Theravāda Buddhist tradition distinguishes between a “path” moment and the immediately subsequent “fruition” state, which can later be repeated and cultivated. As Khema (1994) describes it, “the path moment doesn't have any thinking or feeling in it… While the meditative absorptions bring with them a feeling of oneness, of unity, the path moment does not even contain that. The moment of fruition, subsequent to the path moment, is the understood experience and results in a turned-around vision of existence… The new understanding recognizes every thought, every feeling as stress (*dukkha*)” (Khema, 1994). Of particular note here is how the “fruition” attainment (*phala-samāpatti*) of experiencing or “seeing” cessation that results from insight practice is sharply distinguished by these Theravâda teachers from various results of concentration, including the cessation of consciousness characteristic of the concentrative absorption *nirodha samāpatti* (Griffiths, 1986), as well as from secular goals of stress reduction, and also from other Buddhist conceptions. For instance, according to some schools of Tibetan Buddhism, an enlightened awareness “sees” all phenomena as truly beyond suffering, as an inseparable emptiness-luminosity-bliss state, not different in nature from awareness itself.

Given the differences between various competing conceptions of awakening, one scientific approach to tracing enlightenment would be to use the tools of social psychology to investigate which states and traits are valued in a particular community. For instance, recent work in moral psychology suggests how value judgments of people and practices as either enlightened or unenlightened could be traced to affective reactions of admiration and disgust (Rozin et al., 1999; Schnall et al., 2008; Brandt and Reyna, 2011; Schnall and Roper, 2011). Some of the most virulent disagreements over what counts as genuine awakening occur between closely related practice traditions, such as the debates between various Theravāda Buddhist traditions in Burma over which states are to count as realizations of *nibbāna* and which are instead to be counted (merely) as states of deep concentration. Surveying these debates, Sharf (1995) concludes “there is no public consensus as to the application of terms that supposedly refer to discreet experiential states within the *vipassanā* movement” (Sharf, 1995, p. 265).

It is important in this regard to separate two sorts of disagreement, the one centering on which states are taken to be worthy of the title of awakening, the other on the accuracy of self-reports of experiential states that may reflect markers of progress. Even within communities where there is consensus on which states/traits deserve to be called enlightened, there can be disagreement over whether or not particular individuals have attained these. In practice, as Sharf (1995) details, Buddhist teachers do not accept self-reports of meditative experience at face value, but instead assess whether a particular reported state should be counted as a case of realization depending on practice history, the manner and emotional state with which the report is given, and retrospective observations of behavior. This kind of skepticism is emphasized in Buddhist theory (Olendzki, 2010) and also recent cognitive science (Clark, 1997; Knauper and Turner, 2003; Mehling et al., 2009) suggesting unreliability of self-report, due to affective biases of attention and memory, as well as poor validity and generalizability. Fortunately, objective measures of such affective biases of perception and cognition have been developed (Elliott et al., 2010), and recent work suggests that certain forms of meditation practice may function precisely by attenuating such biases (Vago and Nakamura, 2011; Garland et al., 2012; Van Vugt et al., 2012; Kang et al., 2013). This leads us to a qualified optimism for studies drawing on a nuanced understanding of the specific traditional context of a specific conception of enlightenment and leveraging objective measures of experiential acuity.

## A qualified yes: recent progress and methodological challenges

By integrating evidence from neuroimaging with evidence of behavioral transformations specified in particular traditional descriptions of meditation practices, some important obstacles may be mitigated. For instance, in an adaptation of the Mahasi method developed by Shinzen Young, practitioners use the label “Gone” to refer to the “fruition” experience of cessation described above. According to Young, this kind of experience is not uncommon for advanced practitioners (Young, 2013). Indeed, in a recent study conducted with adept practitioners of this system, two subjects reported having a temporary experience of cessation while in the scanner environment. The methodology was unique in this experiment given that button presses were used to indicate temporal markers associated with peak level of clarity or contact with a sustained period of “rest” that follows ordinary experience of a particular sensory object passing or vanishing from conscious awareness. Using traditional methods of fMRI analyses, we were able to investigate the functional correlates of the deeper experiences of cessation in comparison to the more common experience of the passing away of a sensory object. The preliminary results suggest a number of very unique functional changes in particular brain regions that were similar in activation for “rest” in the other meditators, but the magnitude of the hemodynamic change from baseline was much larger. For example, the frontal polar cortex (Brodmann area-10), a specialized area for higher cognitive functions (Koechlin et al., 1999; Ramnani and Owen, 2004), showed dramatic increases in functional activity (>50% BOLD signal change) that were not as large for the other meditators. As interesting as this preliminary finding is, we can not simply say we found the neural correlate of cessation, but rather a potential neural marker for the experience of “Gone” in Young's system of training that is relative to the baseline state of mind wandering in this individual. Because of the limitations associated with traditional analyses, we are currently attempting to explore this state using more novel methods that do not require the state of interest to be contrasted with a state of no interest, such as state space analyses [see Janoos et al. (2011)].

One major problem in using neuroimaging methodology is that it is typically dependent upon the general linear model (GLM), a convenient method that flexibly allows for both linear regression and ANOVA; however, the model relies on assumptions that may not hold in all situations. For example, fMRI has a poor temporal resolution limited by a hemodynamic response function for each brain response or “state” that is approximately 12 s in duration; any attempts at exploring discrete states within this timeframe is likely to be influenced by “bleeding over” of hemodynamic activity from the previous state. Furthermore, another limitation of the GLM is its dependence on creating a contrast between states of interest and some other state of no interest. The assumptions and limitations are likely to fail in attempts to capture the subtle changes associated with normative, but transitory, states of enlightenment.

Nonetheless, important progress has been made. Some studies investigating long-term meditators with mixed traditions have attempted to map correlational neurophysiology with first-person experiences of clarity, somatic awareness, non-duality, and mind-wandering in adept meditators (Lutz et al., 2004; Josipovic et al., 2012; Garrison et al., 2013; Hagerty et al., 2013; Kerr et al., 2013; Vago et al., 2013). Indeed, neural correlates of particular meditative states show a developmental trajectory, such that there is a similar pattern of development regardless of cultural background or technique early on in meditative naïve practitioners, but that such markers change throughout expertise and experience (e.g., Brefczynski-Lewis et al., 2007). More recently, attempts have been made to operationalize meditation in very specific contexts of automaticity (Travis, 2011) and non-duality (Josipovic, 2010). Some studies have focused on expert practitioners with the assumption that unique neurophysiology amongst these meditators (in comparison to meditation naïve control subjects) may point to particular biomarkers of advancement toward end-goals of practice. For example, Lutz et al. (2004) found gamma band electroencephalography (EEG) power over lateral frontal and parietal electrode sites to correlate (*r* = 0.69) with self-reported clarity in expert Tibetan practitioners of “non-referential compassion” (Tibetan: *dmigs med snying rje*), suggesting a particular mechanism for increased phenomenal intensity. More recently, a number of studies have also found gamma band activity across different electrode sites to correlate with particular meditative states across different contemplative traditions (Lehmann et al., 2001; Vialatte et al., 2009; Cahn et al., 2010, 2013; Rubik, 2011; Berkovich-Ohana et al., 2012; Ferrarelli et al., 2013; Kozhevnikov et al., 2013), some suggesting increased gamma power correlates with level of experience and may be a marker of plasticity that remains during restful or even states of deep sleep (Ferrarelli et al., 2013).

## Conclusions

Scientific investigation of mindfulness and meditation have already arguably advanced the field of neuroscience and specifically our knowledge of the brain (e.g., “resting states”) in the context of functional neuroimaging [see Holzel et al. (2011); Vago and Silbersweig (2012) for review]. Once biomarkers are established for progress along the paths outlined in particular traditions, these can be used as feedback (e.g., Garrison et al., 2013), or for therapeutic targets in the context of neuropsychiatry. It is therefore, necessary to responsibly unpack traditional constructs into common psychological and neurocognitive terms that can correlate with first-person experience with some consistency, but without unwittingly dismissing the deepest and most fundamental features of the practices from which they originate. We are, in the end, cautiously optimistic that progress can be made on well-defined projects in this area that integrate behavior and phenomenology with neuroimaging evidence, but not without a careful consideration of the methodological obstacles. Responsible scientific investigations of enlightenment can proceed only on the basis of rigorous understanding of particular experiential states or behavioral traits within a particular tradition as part of a whole value system, embedded in many other aspects of the models employed in that specific tradition of how the mind works and how awakening progresses.

## References

[B1] Berkovich-Ohana A. Glicksohn J. Goldstein A. (2012). Mindfulness-induced changes in gamma band activity—implications for the default mode network, self-reference and attention. Clin. Neurophysiol. 123, 700–710 10.1016/j.clinph.2011.07.048 21940201

[B2] Brandt M. J. Reyna C. (2011). The chain of being a hierarchy of morality. Perspect. Psychol. Sci. 6, 428–446 10.1177/1745691611414587 26168195

[B3] Brefczynski-Lewis J. A. Lutz A. Schaefer H. S. Levinson D. B. Davidson R. J. (2007). Neural correlates of attentional expertise in long-term meditation practitioners. Proc. Natl. Acad. Sci. U.S.A. 104, 11483–11488 10.1073/pnas.0606552104 17596341 PMC1903340

[B4] Cahn B. R. Delorme A. Polich J. (2010). Occipital gamma activation during Vipassana meditation. Cogn. Process. 11, 39–56 10.1007/s10339-009-0352-1 20013298 PMC2812711

[B5] Cahn B. R. Delorme A. Polich J. (2013). Event-related delta, theta, alpha and gamma correlates to auditory oddball processing during Vipassana meditation. Soc. Cogn. Affect. Neurosci. 8, 100–111 10.1093/scan/nss060 22648958 PMC3541491

[B6] Clark D. A. (1997). Twenty years of cognitive assessment: current status and future directions. J. Consult. Clin. Psychol. 65, 996–1000 10.1037/0022-006X.65.6.996 9420360

[B7] Elliott R. Zahn R. Deakin J. W. Anderson I. M. (2010). Affective cognition and its disruption in mood disorders. Neuropsychopharmacology 36, 153–182 10.1038/npp.2010.77 20571485 PMC3055516

[B8] Ferrarelli F. Smith R. Dentico D. Riedner B. A. Zennig C. Benca R. M. (2013). Experienced mindfulness meditators exhibit higher parietal-occipital eeg gamma activity during nrem sleep. PLoS ONE 8:e73417 10.1371/journal.pone.0073417 24015304 PMC3756031

[B9] Garrison K. A. Santoyo J. F. Davis J. H. Thornhill T.a.T. Kerr C. E. Brewer J. A. (2013). Effortless awareness: using real time neurofeedback to investigate correlates of posterior cingulate cortex activity in meditators' self-report. Front. Hum. Neurosci. 7:440 10.3389/fnhum.2013.00440 23964222 PMC3734786

[B9a] Garland E. L. Boettiger C. A. Gaylord S. Chanon V. W. Howard M. O. (2012). Mindfulness is inversely associated with alcohol attentional bias among recovering alcohol-dependent adults. Cognit. Ther. Res. 36, 441–450 10.1007/s10608-011-9378-7 23280000 PMC3532517

[B10] Griffiths P. J. (1986). On Being Mindless: Buddhist Meditation and the Mind-Body Problem. La Salle: Open Court

[B11] Hagerty M. R. Isaacs J. Brasington L. Shupe L. Fetz E. E. Cramer S. C. (2013). Case study of ecstatic meditation: fMRI and EEG evidence of self-stimulating a reward system. Neural Plast. 2013, 12 10.1155/2013/653572 23738149 PMC3659471

[B12] Holzel B. Lazar S. Gard T. Schuman-Olivier Z. Vago D. Ott U. (2011). How does mindfulness meditation work? Proposing mechanisms of action from a conceptual and neural perspective. Perspect. Psychol. Sci. 6, 537–559 10.1177/1745691611419671 26168376

[B13] Janoos F. Machiraju R. Singh S. Morocz I. A. (2011). Spatio-temporal models of mental processes from fMRI. Neuroimage 57, 362–377 10.1016/j.neuroimage.2011.03.047 21440069

[B14] Josipovic Z. (2010). Duality and nonduality in meditation research. Conscious. Cogn. 19, 1119–1121 10.1016/j.concog.2010.03.016 20385506

[B15] Josipovic Z. Dinstein I. Weber J. Heeger D. J. (2012). Influence of meditation on anti-correlated networks in the brain. Front. Hum. Neurosci. 5:183 10.3389/fnhum.2011.00183 22287947 PMC3250078

[B15a] Kang Y. Gray J. R. Dovidio J. F. (2013). The nondiscriminating heart: lovingkindness meditation training decreases implicit intergroup bias. J. exp. psychol. Gen. [Epub ahead of print]. 10.1037/a0034150 23957283

[B16] Kerr C. E. Sacchet M. D. Lazar S. W. Moore C. I. Jones S. R. (2013). Mindfulness starts with the body: somatosensory attention and top-down modulation of cortical alpha rhythms in mindfulness meditation. Front. Hum. Neurosci. 7:12 10.3389/fnhum.2013.00012 23408771 PMC3570934

[B16a] Khema A. (1994). All of us: beset by birth, decay, and death Nun's Island, Sri Lanka. Available online at: http://www.accesstoinsight.org/lib/authors/khema/allofus.html

[B17] Knauper B. Turner P. A. (2003). Measuring health: improving the validity of health assessments. Qual. Life Res. 12(Suppl. 1), 81–89 10.1023/A:1023589907955 12803314

[B18] Koechlin E. Basso G. Pietrini P. Panzer S. Grafman J. (1999). The role of the anterior prefrontal cortex in human cognition. Nature 399, 148–151 10.1038/20178 10335843

[B19] Kozhevnikov M. Elliott J. Shephard J. Gramann K. (2013). Neurocognitive and somatic components of temperature increases during g-tummo meditation: legend and reality. PLoS ONE 8:e58244 10.1371/journal.pone.0058244 23555572 PMC3612090

[B20] Lehmann D. Faber P. L. Achermann P. Jeanmonod D. Gianotti L. R. Pizzagalli D. (2001). Brain sources of EEG gamma frequency during volitionally meditation-induced, altered states of consciousness, and experience of the self. Psychiatry Res. 108, 111–121 10.1016/S0925-4927(01)00116-0 11738545

[B21] Lutz A. Dunne J. D. Davidson R. J. (2007). Meditation and the neuroscience of consciousness, in Cambridge Handbook of Consciousness, eds Zelazo P. Moscovitch M. Thompson E. (New York, NY: Cambridge University Press), 499–555

[B22] Lutz A. Greischar L. L. Rawlings N. B. Ricard M. Davidson R. J. (2004). Long-term meditators self-induce high-amplitude gamma synchrony during mental practice. Proc. Natl. Acad. Sci. U.S.A. 101, 16369–16373 10.1073/pnas.0407401101 15534199 PMC526201

[B23] Mehling W. E. Gopisetty V. Daubenmier J. Price C. J. Hecht F. M. Stewart A. (2009). Body awareness: construct and self-report measures. PLoS ONE 4:e5614 10.1371/journal.pone.0005614 19440300 PMC2680990

[B24] Olendzki A. (2010). Vipallasa Sutta: Distortions of the Mind [Online]. Available online at: http://www.accesstoinsight.org/tipitaka/an/an04/an04.049.olen.html

[B25] Ramnani N. Owen A. M. (2004). Anterior prefrontal cortex: insights into function from anatomy and neuroimaging. Nat. Rev. Neurosci. 5, 184–194 10.1038/nrn1343 14976518

[B26] Rozin P. Lowery L. Imada S. Haidt J. (1999). The CAD triad hypothesis: a mapping between three moral emotions (contempt, anger, disgust) and three moral codes (community, autonomy, divinity). J. Pers. Soc. Psychol. 76, 574–586 10.1037/0022-3514.76.4.574 10234846

[B27] Rubik B. (2011). Neurofeedback-enhanced gamma brainwaves from the prefrontal cortical region of meditators and non-meditators and associated subjective experiences. J. Altern. Complement. Med. 17, 109–115 10.1089/acm.2009.0191 21303197

[B28] Sayadaw M. (1992). On the Nature of Nibbāna. Mayasia: Subang Jaya Buddhist Association

[B29] Schnall S. Haidt J. Clore G. L. Jordan A. H. (2008). Disgust as embodied moral judgment. Pers. Soc. Psychol. Bull. 34, 1096–1109 10.1177/0146167208317771 18505801 PMC2562923

[B30] Schnall S. Roper J. (2011). Elevation puts moral values into action. Soc. Psychol. Pers. Sci. 3, 373–378 10.1177/1948550611423595

[B31] Sharf R. H. (1995). Buddhist modernism and the rhetoric of meditative experience. Numen 42, 228–283 10.1163/1568527952598549

[B32] Tang Y. Y. Lu Q. Geng X. Stein E. A. Yang Y. Posner M. I. (2010). Short-term meditation induces white matter changes in the anterior cingulate. Proc. Natl. Acad. Sci. U.S.A. 107, 15649–15652 10.1073/pnas.1011043107 20713717 PMC2932577

[B33] Travis F. (2011). Comparison of coherence, amplitude, and eLORETA patterns during Transcendental Meditation and TM-Sidhi practice. Int. J. Psychophysiol. 81, 198–202 10.1016/j.ijpsycho.2011.06.011 21726586

[B34] Vago D. R. Nakamura Y. (2011). Selective attentional bias towards pain-related threat in fibromyalgia: preliminary evidence for effects of mindfulness meditation training. Cogn. Ther. Res. 35, 581–594 10.1007/s10608-011-9391-x

[B35] Vago D. R. Pan H. Silbersweig D. A. Stern E. (2013). Neural substrates underlying modalities of awareness in mindfulness practice, in American Neuropsychiatric Assocation Annual Meeting (Boston, MA). 10.1111/nyas.12270

[B36] Vago D. R. Silbersweig D. A. (2012). Self-awareness, self-regulation, and self-transcendence (S-ART): a framework for understanding the neurobiological mechanisms of mindfulness. Front. Hum. Neurosci. 6:296 10.3389/fnhum.2012.00296 23112770 PMC3480633

[B38] Van Vugt M. K. Hitchcock P. Shahar B. Britton W. (2012). The effects of mindfulness-based cognitive therapy on affective memory recall dynamics in depression: a mechanistic model of rumination. Front. Hum. Neurosci. 6:257 10.3389/fnhum.2012.00257 23049507 PMC3446543

[B39] Vialatte F. B. Bakardjian H. Prasad R. Cichocki A. (2009). EEG paroxysmal gamma waves during Bhramari Pranayama: a yoga breathing technique. Conscious. Cogn. 18, 977–988 10.1016/j.concog.2008.01.004 18299208

[B40] Xue S. Tang Y. Y. Posner M. I. (2011). Short-term meditation increases network efficiency of the anterior cingulate cortex. Neuroreport 22, 570–574 10.1097/WNR.0b013e328348c750 21691234

[B41] Young S. (2013). 5 Ways to Know Yourself: An Introduction to Basic Mindfulness [Online]. Available online at: http://www.shinzen.org/Retreat%20Reading/FiveWays.pdf

[B42] Zeidan F. Johnson S. K. Diamond B. J. David Z. Goolkasian P. (2010). Mindfulness meditation improves cognition: evidence of brief mental training. Conscious. Cogn. 19, 597–605 10.1016/j.concog.2010.03.014 20363650

